# The word-with-noise test: development, validation and reference values

**DOI:** 10.1590/2317-1782/20242023091en

**Published:** 2024-05-31

**Authors:** Lidiéli Dalla Costa, Ana Valéria de Almeida Vaucher, Karina Carlesso Pagliarin, Maristela Julio Costa

**Affiliations:** 1 Universidade Federal de Santa Maria – UFSM - Santa Maria (RS), Brasil.; 2 Hospital Universitário de Santa Maria – HUSM - Santa Maria (RS), Brasil.

**Keywords:** Hearing, Speech Perception, Hearing Tests, Noise, Validation Study, Psychometrics, Adult

## Abstract

**Purpose:**

To propose an instrument for assessing speech recognition in the presence of competing noise. To define its application strategy for use in clinical practice. To obtain evidence of criterion validity and present reference values.

**Methods:**

The study was conducted in three stages: Organization of the material comprising the Word-with-Noise Test (Stage 1); Definition of the instrument's application strategy (Stage 2); Investigation of criterion validity and definition of reference values for the test (Stage 3) through the evaluation of 50 normal-hearing adult subjects and 12 subjects with hearing loss.

**Results:**

The Word-with-Noise Test consists of lists of monosyllabic and disyllabic words and speech spectrum noise (Stage 1). The application strategy for the test was defined as the determination of the Speech Recognition Threshold with a fixed noise level at 55 dBHL (Stage 2). Regarding criterion validity, the instrument demonstrated adequate ability to distinguish between normal-hearing subjects and subjects with hearing loss (Stage 3). Reference values for the test were established as cut-off points expressed in terms of signal-to-noise ratio: 1.47 dB for the monosyllabic stimulus and -2.02 dB for the disyllabic stimulus. **Conclusion:** The Word-with-Noise Test proved to be quick to administer and interpret, making it a useful tool in audiological clinical practice. Furthermore, it showed satisfactory evidence of criterion validity, with established reference values.

## INTRODUCTION

The basic audiological assessment consists of a battery of tests, including pure-tone audiometry, speech perception assessment and acoustic immittance measurements^(^1^)^. Although the results obtained through these tests are extremely important and indispensable for audiological diagnosis, their results portray the subject’s auditory performance in a favorable listening situation. However, most everyday communicative situations occur in environments where listening is impaired by the presence of competitive noise^(^2^)^.

This listening condition in noisy environments is unfavorable for speech intelligibility, a difficulty frequently reported by patients at the audiology clinic^(^3^)^. Furthermore, individuals with the same tonal thresholds and the same speech recognition auditory abilities in a silent environment may present extremely different recognition abilities in noisy environments^(^4^)^.

The speech recognition assessment in the presence of competitive noise is considered important and has wide clinical use. However, according to the literature consulted, in Brazil there is a lack of a test with words that can be performed in the presence of noise, with psychometric studies on its development.

Speech recognition tests in noise make it possible to quantify, in a more real and objective manner, each individual’s speech recognition capacity, validating the difficulties reported by them in unfavorable auditory situations^(^5-7^)^. Still, it contributes to the planning of professional conduct and guidance for the subject with this type of complaint.

Therefore, the results of a standardized test to evaluate speech recognition in noise using words are extremely important for audiological diagnosis, especially in cases that do not present impairment in speech recognition in a quiet environment, but there are complaints related to speech recognition in the presence of noise, which can occur, mainly, in mild hearing losses or losses with a descending configuration^(^6^)^. For this reason, it is understood that the proposition of a test that allows the assessment of speech recognition in noise, which is developed for specific use to complement the basic audiological battery, with psychometric measures and a determined application strategy, has proven to be necessary in audiological routine.

Therefore, this study aimed to: propose a speech recognition instrument in the presence of competitive noise; define its application strategy; seek evidence of validity; and establish reference values.

## METHOD

Applied research of an observational analytical cross-sectional quantitative nature was approved by the Research Ethics Committee (CEP) of a Higher Education Institution under no. 3.660.209, meeting all ethical conduct standards, in accordance with the Guidelines and Standards Regulations for Research involving Human Beings (Resolution 466/12 of the National Health Council). All research participants signed the Free Consent Form (FCF).

### Origin of the material used to organize the Word-with-Noise Test

To organize the instrument, lists of monosyllabic^(^8-10^)^ and disyllabic^(^11,12^)^ words and noise with speech spectrum^(^13^)^ previously developed and already published were used, made available by the authors for new material composition.

It is important to highlight that the lists of monosyllabic and disyllabic words were developed under strict criteria, with psychometric studies to validate content, criteria, construct, both for monosyllabic^(^8-10^)^ and disyllabic^(^11,12^)^ lists. Nevertheless, these lists were proposed for application in a quiet environment. The lists of monosyllabic words are called L1 and L2 and the disyllabic ones are called LD-A, LD-B, LD-C, LD-D, LD-E, and each list consists of 25 words. These word lists were digitally recorded in a studio, in accordance with ISO 8253-3:2012, with a female speaker’s voice.

Regarding the speech spectrum noise used in this study, it was developed specifically to be applied in the speech recognition assessment in the presence of competitive noise, in a clinical situation^(^13^)^.

This study was carried out in three stages: Stage 1 – Organization of the material; Stage 2 – Instrument application strategy (standardization); Stage 3 – Criterion validity and reference values.

#### Stage 1: Organization of the material

To organize the new assessment material, a digital edition of the word lists and noise was carried out by a professional sound technician and audio operator, at a recording studio, who used the original recordings of the materials, which gave rise to new digital material, with the content of the proposed test.

Initially, to prepare and record the material digitally, the different stimuli were adjusted, that is, pure tone, speech and noise, in order to ensure that they were at the same recording level.

#### Stage 2: Instrument application strategy

From the organization of the test material, it was initially applied to five normal-hearing adults, in order to verify its applicability and define the application protocol. At this stage, different strategies were used, and Speech Recognition Thresholds (SRTs) and Speech Recognition Percentage Indexes were researched, using different presentation levels of speech and noise stimuli.

#### Stage 3: Criterion validity and reference values

##### Participants

The sample for this study was taken by convenience. Normal-hearing participants were recruited through an invitation published on social networks and a verbal invitation from the researcher. For the selection and recruitment of participants with hearing loss, a search was carried out on the database of the Hearing Aids Laboratory of the Higher Education Institution, seeking to select participants according to the eligibility criteria.

The inclusion criteria for the normal-hearing group were: being between 19 and 44 years old; airway hearing thresholds lower than 20 dBHL at frequencies from 250 to 8000 Hz; having at least completed primary education, and being right-handed (confirmed based on the Edinburgh Handedness Inventory)^(^14,15^)^. The following exclusion criteria were defined: presenting hearing complaints; middle ear changes; complaints and/or neuropsychiatric signs and symptoms, and/or noticeable speech changes.

Considering these criteria, 50 normal-hearing subjects participated in the research, where 39 were women (78%) and 11 were men (22%), aged between 19 and 40 years, with an average age of 25.5 years.

For the group of participants with hearing loss, the following inclusion criteria were considered: subjects over 19 years old; conductive, sensorineural or mixed hearing loss^(^16^)^; average audibility thresholds for frequencies of 500, 1000, 2000, and 4000 Hz, ranging from mild to moderate hearing loss^(^17^)^; presentation of a Speech Recognition Percentage Index (SRPI) result in a quiet environment between 100% and 80%; report of auditory complaints of difficulty recognizing speech in noise; having at least completed primary education and being right-handed (confirmed based on the Edinburgh Handedness Inventory)^(^14,15^)^. The following exclusion criteria were established: complaints and/or neuropsychiatric signs and symptoms; cognitive changes (screened using the Mini Mental State Examination)^(^18,19^)^ and/or noticeable speech changes.

Twelve subjects with hearing loss participated in the study, five women (41.67%) and seven men (58.33%), between 38 and 70 years old, and an average of 58.42 years old. Of these subjects, one ear presented conductive hearing loss (4.76%), 17 ears presented sensorineural hearing loss (80.95%), and three ears presented mixed hearing loss (14.29%). Regarding the hearing loss degree, nine ears presented mild loss (42.86%), and 12 ears presented a moderate degree (57.14%).

##### Instruments and procedures

Participants underwent targeted anamnesis, with questions regarding personal data, education level, otological history, and hearing complaints. Subsequently, a visual inspection of the External Acoustic Meatus (EAM) of both ears was carried out, an assessment of acoustic immittance measurements, Tonal Threshold Audiometry (TTA), speech perception assessment, and finally they were evaluated with the instrument proposed in this study.

Acoustic immittance measurements were carried out using the Interacoustics AT 235 tympanometer. The TTA and the application of the proposed assessment instrument were performed using the Interacoustics AC 33 audiometer, and TDH 39 earphones, in an acoustically treated environment. In addition, one also used a Toshiba CD-4149 Player coupled to the audiometer to present speech and noise stimuli in digital recording. The average duration of the complete assessment for each subject was 60 minutes.

##### Data analysis

To investigate criterion validity and determine reference values for the Word-with-Noise (WIN) Test, the Receiver Operating Characteristic (ROC) curve technique was used.

Initially, a comparison was made between the performances of the evaluated ears (right and left) of the normal-hearing subjects. No statistically significant differences were observed in the ear variable for monosyllabic (p=0.463) and disyllabic (p=0.295) words using the Wilcoxon test.

Subsequently, performance was compared between female and male normal-hearing subjects. There were also no statistically significant differences in the gender variable for monosyllabic (p=0.242) and disyllabic (p=0.171) words in the Mann-Whitney test.

These analyses allowed reference values to be generated considering the general result of the ears, totaling 100 ears evaluated, generating a single cut-off point independent of these variables, in order to facilitate the interpretation of the results.

Statistical analyses were carried out using SPSS V20, Minitab 16 and Excel Office 2010. A significant result was considered p ≤ 0.05, with a 95% confidence interval.

## RESULTS

### Stage 1: Resulting material

The instrument proposed in this study was called the Word-with-Noise (WIN) Test. The test content includes a track with the 1 KHz reference signal (pure tone) and the noise with speech spectrum for calibration purposes; a track with the introductory test instruction phrase; a monosyllabic training list; two equivalent monosyllabic lists, presented with two sequences of different word distributions, and five equivalent monosyllabic lists.

Considering that there are only two lists of monosyllabic words, and seeking to avoid the memory/learning effect in different applications, two different word distribution sequences were made available, forming four lists (L1 sequence 1 and L1 sequence 2; L2 sequence 1 and L2 sequence two).

The word lists and noise were recorded on independent channels, thus allowing presentation levels on each channel to be adjusted in isolation.

The introductory sentence for running the test in noise is as follows: “You will hear a list of words and a noisy sound. Ignore the noise and repeat each word you hear as you understand it”.

In all lists, words are preceded by the command “repeat the word”.

### Stage 2: Selected instrument application strategy

Based on the observation of the different application strategies performed on the five individuals, data analysis and participants’ reports after carrying out the test and also, based on the literature, the WIN Test application strategy in this research was chosen to obtain the SRT in the presence of fixed noise.

To obtain the measurements, the WIN Test was applied by using earphones, monaurally, with the speech stimulus being presented together with the noise. In order for the presentation level of the two stimuli to be adjusted independently, they were recorded on different channels. Therefore, before starting the test application, the equipment was calibrated using the VU-meter, adjusting the output of each channel to zero level, having as a reference a pure 1 kHz tone present on the same channel on which the words were recorded, while on the other channel the test noise itself was used, as it is continuous noise.

The monosyllabic and disyllabic lists were applied in a randomized manner, and the measurements were obtained taking care to evaluate the ears alternately, classifying the subjects into *odd* and *even*, and thus the assessment of the even subjects was initiated by the right ear, and the assessment of the odd subjects by the left ear.

Consequently, the WIN Test was applied according to the following order of presentation:

1. Independent calibration of each channel of the equipment;
2. Instruction on the test response strategy in noise on the side of the selected ear to start the test;
3. Application of the training list presenting the first 10 monosyllabic words, with the presence of competitive noise, to familiarize the subject with the test, alternating ears;
4. Application of a list of monosyllables, in the presence of competitive noise, alternating the side of the ear again;
5. Application of a list of disyllables, in the presence of competitive noise, maintaining the side of the ear previously assessed;
6. Application of a list of monosyllables, in the presence of competitive noise, alternating the side of the ear;
7. Application of a list of disyllables, in the presence of competitive noise, maintaining the ear side previously assessed.

To research the SRT using the WIN Test, the sequential or adaptive – or even ascending-descending^(^20^)^ – strategy was used, which makes it possible to determine the condition in which the individual is able to recognize around 50% of the speech signals heard.

To begin analyzing the SRT in noise, an S/N ratio of + 10 dBHL was used and the training list was presented. Based on this, the test started being applied, with the presentation of the first list word at a level of 10 dBHL above the value of the first error made during the application of the training list, which ensures that the subject starts getting the first list word correct, thus seeking to reduce the variability of measurements and also serving as a motivator for the subject under evaluation. Subsequently, the next word was presented at a level of 4 dBHL below and so on, until the subject evaluated presented the first incorrect answer, and from then on, presentation intervals of 2 dBHL were used until the end of the list, according to the subject’s response, that is, when the response was incorrect, the level was increased by 2 dB, and when it was correct, it was decreased by 2 dB^(^20^)^.

The requested response strategy was to repeat the words in the way they understood, and in cases in which the subject answered two similar words due to having doubts, the first repeated word was considered.

To obtain the SRTs, the presentation levels of all words were noted and the means of these values were calculated from the value at which the first response reversal occurred (first error), until the end of the list.

Considering that an S/N ratio is obtained by calculating the difference, in dB, between the SRT value (average of speech presentation levels in the presence of noise) and the value of the competitive noise used, this calculation was carried out based on the values obtained for each list, subtracted from the noise presentation level used in this research, which was 55 dBHL.

The estimated application time of the WIN Test is 30 seconds for calibration, 11 seconds to execute the instruction phrase, around 1 minute and 30 seconds to apply the training list, 3 minutes and 22 seconds to execute the monosyllabic list in each ear, and 3 minutes and 20 seconds to apply the disyllabic lists in each ear. Therefore, the approximate total time to perform the test in both ears with monosyllables or disyllables is 9 minutes, and if necessary, 16 minutes to apply the associated monosyllabic and disyllabic lists.

### Stage 3: Criterion validity and reference values

In Table 1, a comparison can be seen between the performance of the ears of normal-hearing subjects and the ears of subjects with hearing loss in the WIN Test, in the assessment with monosyllabic and disyllabic words. For both the monosyllabic and disyllabic stimuli, statistically significant differences were found between the groups evaluated, with better performance being observed in the normal-hearing group. It is also possible to observe that both normal-hearing subjects and subjects with hearing loss performed better when evaluated with disyllabic words.

**Table 1:** Comparison of performance by ear between normal-hearing subjects and those with hearing loss in the Word-with-Noise Test

		**N**	**Mean**	**Median**	**Standard deviation**	**Q1**	**Q3**	**CI**	**P-value**
Monosyllables	Normal-hearing subjects	100	-2.38	-2.39	1.86	-3.82	-1.17	0.36	<0.001*
	Hearing loss	21	6.44	6.17	4.31	3.78	9.83	1.85	
Disyllables	Normal-hearing subjects	100	-5.99	-6.15	1.63	-7.23	-4.70	0.32	<0.001*
	Hearing loss	21	1.61	0.81	2.70	-0.36	4.14	1.16	

Mann-Whitney test. Results expressed in Signal/Noise ratio

* Statistically significant value at the 5% level (p ≤ 0.05)

**Legend:** N: number of ears; Q: quartile; CI: confidence interval

In the analysis of the ROC curve, it was found that both curves are statistically significant with p-value <0.001 and extremely high *Area Under the Curve* (AUC) values, they are 0.987 and 1.000, respectively, for the monosyllabic and disyllabic stimuli.

**Figure 1:**
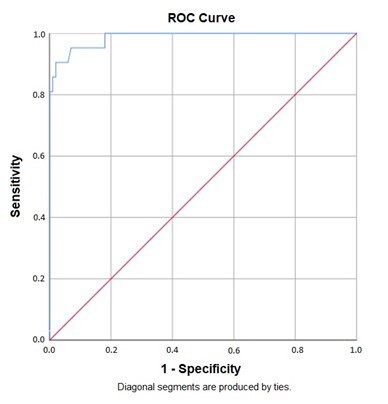
ROC Curve for the Word-with-Noise Test with Monosyllables

**Figure 2:**
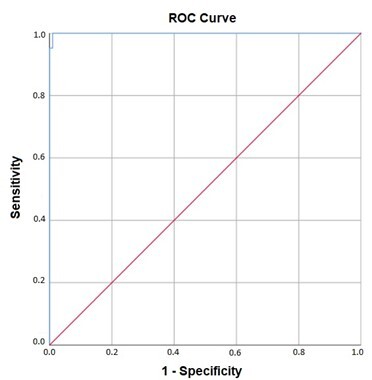
ROC Curve for the Word-with-Noise Test with Disyllables

The ROC curves for monosyllables and disyllables are shown in Figures 1 and 2, respectively.

Table 2 shows the cut-off points for monosyllabic and disyllabic WIN Test. The ideal cut-off points were determined by finding the values that allowed the best balance between sensitivity and specificity.

**Table 2:** Word-with-Noise Test cut-off score obtained using ROC curves, and corresponding sensitivity and specificity

	**Cut-off point**	**Sensitivity**	**specificity**
Monosyllables	1.47	90.5%	98.0%
Disyllables	-2.02	100.0%	99.0%

Cut-off point expressed in Signal/Noise ratio

## DISCUSSION

The WIN Test proposal arose from the need to complement the basic audiological battery, with a speech recognition test in noise that is quick and easy to apply, with well-defined psychometric characteristics. The test is intended to investigate one of the main auditory complaints reported by subjects when carrying out hearing assessments in clinical practice, which is the difficulty recognizing speech in noisy environments^(^3^)^.

The WIN Test application strategy defined for this research, which was the Speech Recognition Threshold (SRT) research using fixed noise at 55 dBHL sought to represent a communicative situation commonly experienced in everyday life.

The SRT, when presented with competitive noise, is capable of measuring speech recognition performance and establishing the S/N ratio necessary for the individual to repeat correctly 50% of the speech stimuli presented in noise^(^21^)^.

According to the report from the World Health Organization^(^22^)^, environmental noise measures above 55 dB(A) can already cause mild stress, triggering behaviors such as hearing discomfort, a state of vigilance and agitation, which, in turn, can cause changes in the results of a subjective test. This information reinforces the choice of presentation level used in this research, aiming to minimize the tiredness and auditory discomfort of the subject evaluated.

This assessment strategy is widely applied in international tests that assess speech recognition in noise using words as stimuli, such as the Words-in-Noise Test (WIN)^(^23^)^, Digits in Noise^(^24^)^, and the Speech in Babble (SiB)^(^25^)^. It is also recommended by professional guidelines, which consider the adaptive SRT research procedure in noise as the most suitable for use in the basic audiological assessment battery, which provides a quick and standardized measurement^(^26^)^.

The main advantage of using SRT as a strategy for evaluating speech recognition in noise is the fact that the presentation level of the speech stimulus is adaptable according to each subject’s response, thus avoiding test ceiling effect and floor effect, which are related to extremely higher (100%) and lower (0%) scores, respectively^(^27^)^.

Regarding the comparison between the WIN Test performance of normal-hearing subjects and subjects with hearing loss and complaints of difficulty recognizing speech in noise, better performance was observed in normal-hearing subjects, in the assessment with both monosyllabic and disyllabic stimuli (Table 1), demonstrating criterion validity. This finding is in line with other studies that evaluated speech recognition in noise through tests with words as well^(^3,28^)^ and also observed better performance in normal-hearing subjects who did not present complaints related to speech recognition in noise. Furthermore, it was possible to notice that both groups performed better when evaluated with disyllabic words (Table 1). This result was expected, as the longer a word is, the easier it is to be recognized^(^29^)^.

The analysis of the ROC curve revealed satisfactory evidence of criterion validity through extremely high AUC values (Figures 1 and 2), which means that the WIN Test presented adequate ability to distinguish between normal-hearing subjects and those without complaints of difficulty recognizing speech in noise and subjects with hearing loss and complains of difficulty recognizing speech in noise.

Regarding the reference values (cut-off point) established for the WIN Test, the results of the sensitivity and specificity analysis (Table 2) indicated an S/N ratio of 1.47 for WIN-M (90.5% sensitivity, 98.0% specificity) and -2.02 for WIN-D (100.0% sensitivity, 99.0% specificity). For this study, sensitivity was considered the test ability to identify subjects with difficulty recognizing speech in noise, while specificity was considered the test ability to express results within normal limits, for those subjects who do not have difficulty recognizing speech in noise.

Therefore, performances at more favorable (more positive) S/N ratios than these values demonstrate that the subject being assessed has difficulty recognizing speech in noise and indicate the need to use other materials and complementary tests for a more detailed assessment. These tests should allow us to better measure how much these altered results predict the communicative difficulties faced on a daily basis by a given individual, such as, for example, assessment with sentence stimuli and/or assessment of auditory processing skills.

Regarding the stimulus to be used to evaluate speech recognition in noise, based on the observations made in this study, it is believed that both stimuli presented here (monosyllabic and disyllabic words) can be used, but it is important to consider that each will provide different information and can even be used together to better elucidate the individual’s ability to recognize speech in noise in clinical routine.

It is known that monosyllabic words, despite carrying linguistic context, are more related to audibility. Disyllabic words, on the other hand, carry a greater linguistic context and provide a greater number of auditory clues, which, as can be confirmed in this research, facilitates their recognition, in more unfavorable S/N listening relationships, and are also more representative of the daily routine, due to the greater number of disyllables in the Brazilian Portuguese language.

Thus, considering this, it can be suggested that the evaluator preferentially applies monosyllabic lists, and then, when the subject presents unsatisfactory performance (or below expectations), a list of disyllables is applied with the aim of verifying how much the individual is able to take advantage of the increase in semantic and linguistic clues, provided by the increase in the word length, in the task of recognizing speech^(^30^)^.

For the result of applying the WIN-D associated with WIN-M, an S/N ratio is expected to be at least equal and ideally lower (more challenging) when evaluating subjects with disyllabic words. Failure to take advantage of the increase in auditory and linguistic clues provided by disyllables may indicate changes in different auditory and/or cognitive processing skills, which require more detailed investigation.

The WIN Test demonstrated to be applicable in the audiological clinical routine, fast and easy to use and interpret, in addition to having been developed in the Brazilian Portuguese language, consisting of familiar words, noise with a speech spectrum, and presentation in digital format. It also showed the ability to identify subjects with difficulty recognizing speech in noise, both with monosyllabic and disyllabic stimuli.

Therefore, it is suggested that the WIN Test should be included in the basic audiological assessment battery of those subjects with mild to moderate hearing loss^(^17^)^, or hearing loss with descending configuration, who present adequate or mild difficulty in speech recognition in silence, assessed using conventional speech audiometry, but reporting complaints related to speech recognition in the presence of noise, in order to detect this complaint objectively.

When using the WIN Test, the examiner should be aware of the hearing loss degree and configuration in order to ensure that the noise is being perceived, considering the fact that the noise presentation level in the WIN Test is 55 dBHL, thus ensuring that the assessment is being carried out in the presence of competitive noise.

It should be noted that if the examiner uses different noise levels, or makes any change in the test application strategy, the reference values described in this work will not be valid.

Regarding the limitations of the study, due to the time determined for this research, it was not possible to apply the WIN Test to subjects with more severe hearing losses, which would require some adaptations to the application protocol, and it is a possibility for further studies.

## CONCLUSION

The WIN Test was proposed for use in the basic audiological battery, in order to identify subjects with difficulty recognizing speech in noise. The test application strategy was defined as researching the Speech Recognition Threshold with noise fixed at 55 dBHL. The WIN Test proved to be quick and of easy application and result interpretation and can represent a useful tool to be used in the audiological clinical routine. Furthermore, it presented satisfactory evidence of criterion validity and ability to identify subjects with difficulty recognizing speech in noise, with both monosyllabic and disyllabic stimuli. Cut-off points expressed in the S/N ratio of 1.47 dB for WIN-M and -2.02 dB for WIN-D were defined as reference values.
