# Supplementary material for: The word-with-noise test: development, validation and reference values
Source: Codas. 2024 May 31;36(3):e20230091. doi: 10.1590/2317-1782/20242023091en (PMC467000; doi:10.1590/2317-1782/20242023091en)
Supplement: Translation

# Translation: Teste de palavras no ruído: desenvolvimento, validação e valores de referência

**DOI:** 10.1590/2317-1782/20242023091pt

**Authors:** Lidiéli Dalla Costa, Ana Valéria de Almeida Vaucher, Karina Carlesso Pagliarin, Maristela Julio Costa

**Affiliations:** 1 Universidade Federal de Santa Maria – UFSM - Santa Maria (RS), Brasil.; 2 Hospital Universitário de Santa Maria – HUSM - Santa Maria (RS), Brasil.

**Keywords:** Audição, Percepção da Fala, Testes Auditivos, Ruído, Estudo de Validação, Psicometria, Adulto

## Abstract

**Objetivo:**

Propor um instrumento para a avaliação do reconhecimento de fala na presença de ruído competitivo. Definir sua estratégia de aplicação, para ser aplicado na rotina clínica. Obter evidências de validade de critério e apresentar seus valores de referência.

**Método:**

Estudo realizado em três etapas: Organização do material que compôs o Teste de Palavras no Ruído (Etapa 1); Definição da estratégia de aplicação do instrumento (Etapa 2); Investigação da validade de critério e definição dos valores de referência para o teste (Etapa 3), por meio da avaliação de 50 sujeitos adultos normo-ouvintes e 12 sujeitos com perda auditiva.

**Resultados:**

O Teste de Palavras no Ruído é composto por listas de vocábulos mono e dissilábicos e um ruído com espectro de fala (Etapa 1). Foi definida como estratégia de aplicação do teste, a realização do Limiar de Reconhecimento de Fala com ruído fixo em 55 dBNA (Etapa 2). Quanto à validade de critério, o instrumento apresentou adequada capacidade de distinção entre os sujeitos normo-ouvintes e os sujeitos com perda auditiva (Etapa 3). Foram definidos como valores de referência para o teste, os pontos de corte expressos em relação sinal/ruído de 1,47 dB para o estímulo monossilábico e de -2,02 dB para o dissilábico.

**Conclusão:**

O Teste de Palavras no Ruído demonstrou ser rápido e de fácil aplicação e interpretação dos resultados, podendo ser uma ferramenta útil a ser utilizada na rotina clínica audiológica. Além disso, apresentou evidências satisfatórias de validade de critério, com valores de referência estabelecidos.

## INTRODUÇÃO

A avaliação audiológica básica é composta por uma bateria de testes, dentre eles estão a audiometria tonal liminar, logoaudiometria e medidas de imitância acústica^(1)^. Apesar dos resultados obtidos por meio destes testes serem extremamente importantes e indispensáveis para o diagnóstico audiológico, seus resultados retratam o desempenho auditivo do sujeito em uma situação favorável de escuta. No entanto, grande parte das situações comunicativas cotidianas, ocorre em ambientes cuja escuta é prejudicada pela presença de ruído competitivo^(2)^.

Essa condição de escuta em ambientes ruidosos é desfavorável para a inteligibilidade da fala, uma dificuldade frequentemente relatada por pacientes na clínica audiológica^(3)^. Ainda, indivíduos com os mesmos limiares tonais e com as mesmas habilidades auditivas de reconhecimento de fala em ambiente silencioso, podem apresentar habilidades de reconhecimento extremamente diferentes em ambientes ruidosos^(4)^.

A avaliação do reconhecimento de fala na presença de ruído competitivo é considerada importante e com ampla utilização clínica. Entretanto, de acordo com a literatura consultada, no Brasil, há uma carência de um teste com palavras que seja executado na presença de ruído, com estudos psicométricos sobre a sua elaboração.

Testes de reconhecimento de fala no ruído possibilitam que seja quantificada, de forma mais real e objetiva, a capacidade de reconhecimento de fala de cada indivíduo, validando a dificuldade referida pelo mesmo em situações auditivas desfavoráveis^(5-7)^. E também, contribuindo para o planejamento da conduta profissional e para a orientação do sujeito com este tipo de queixa.

Assim sendo, os resultados de um teste padronizado para avaliação do reconhecimento de fala no ruído utilizando palavras, são de extrema importância para o diagnóstico audiológico, principalmente nos casos que não apresentam prejuízo no reconhecimento de fala em ambiente silencioso, porém referem queixas relacionadas ao reconhecimento de fala na presença de ruído, o que pode ocorrer, principalmente, em perdas auditivas de grau leve ou perdas com configuração descendente^(6)^.

Portanto, entende-se que a proposição de um teste que permita a avaliação do reconhecimento de fala no ruído, que seja desenvolvido para o uso específico na complementação da bateria audiológica básica, com medidas psicométricas e estratégia de aplicação determinadas, tem se mostrado necessária na rotina audiológica.

Desta forma, este estudo teve como objetivos: propor um instrumento de reconhecimento de fala na presença de ruído competitivo; definir sua estratégia de aplicação; buscar evidências de validade; estabelecer valores de referência.

## MÉTODO

Pesquisa aplicada, de natureza analítico observacional, transversal e quantitativa, foi aprovada pelo Comitê de Ética em Pesquisa (CEP) de uma Instituição de Ensino Superior sob número 3.660.209, atendendo todas as normas éticas de conduta, de acordo com as Diretrizes e Normas Regulamentadoras de Pesquisa envolvendo Seres Humanos (Resolução 466/12 do Conselho Nacional de Saúde). Todos os participantes da pesquisa assinaram o Termo de Consentimento Livre e Esclarecido (TCLE).

### Origem do material utilizado para a organização do Teste de Palavras no Ruído

Para a organização do instrumento, foram utilizadas listas de vocábulos mono^(8-10)^ e dissilábicos^(11,12)^ e um ruído com espectro de fala^(13)^ desenvolvidos anteriormente e já publicados, disponibilizados pelas autoras para composição do novo material.

É importante salientar que as listas de vocábulos mono e dissilábicos foram desenvolvidas sob rigorosos critérios, com estudos psicométricos de validação de conteúdo, critério, construto, tanto para as listas mono^(8-10)^ quanto para as dissilábicas^(11,12)^. No entanto, estas listas foram propostas para aplicação em ambiente silencioso. As listas de palavras monossilábicas são denominadas L1 e L2 e as dissilábicas são denominadas LD-A, LD-B, LD-C, LD-D, LD-E, sendo cada lista composta por 25 vocábulos. Essas listas de palavras foram gravadas digitalmente em um estúdio, de acordo com a norma ISO 8253-3:2012, com a voz de um locutor do sexo feminino.

Com relação ao ruído com espectro de fala utilizado neste estudo, ele foi desenvolvido, especificamente para ser aplicado na avaliação do reconhecimento de fala na presença de ruído competitivo, em situação clínica^(13)^.

Este estudo foi realizado em três etapas: Etapa 1 – Organização do material; Etapa 2 – Estratégia de aplicação do instrumento (padronização); Etapa 3 – Validade de critério e valores de referência.

#### Etapa 1: Organização do material

Para a organização do novo material de avaliação, foi realizada uma edição digital das listas de palavras e do ruído, por um profissional técnico de som e operador de áudio, em estúdio de gravação, que utilizou as gravações originais dos materiais, às quais deram origem a um novo material digital, com o conteúdo do teste proposto.

Inicialmente, para a preparação e gravação do material em formato digital, foi realizado o ajuste dos diferentes estímulos: tom puro, fala e ruído, a fim de garantir que estes estivessem no mesmo nível de gravação.

#### Etapa 2: Estratégia de aplicação do instrumento

A partir da organização do material de teste, este foi aplicado inicialmente em cinco adultos normo-ouvintes, a fim de verificar sua aplicabilidade e definir o protocolo de aplicação. Nesta etapa, diferentes estratégias foram usadas, tendo sido pesquisados os Limiares de Reconhecimento de Fala (LRF) e Índices Percentuais de Reconhecimento de Fala, utilizando diferentes níveis de apresentação dos estímulos de fala e ruído.

#### Etapa 3: Validade de critério e valores de referência

##### Participantes

A amostra deste estudo ocorreu por conveniência. Os participantes normo-ouvintes foram recrutados por meio de convite divulgado em redes sociais e convite verbal do próprio pesquisador. Já para seleção e recrutamento dos participantes com perda auditiva foi efetuada uma pesquisa na base de dados do Laboratório de Próteses Auditivas da Instituição de Ensino Superior, buscando selecionar os participantes de acordo com os critérios de elegibilidade.

Os critérios de inclusão para o grupo de normo-ouvintes foram: apresentar idade entre 19 e 44 anos; limiares auditivos de via aérea menores que 20 dBNA nas frequências de 250 a 8000 Hz; possuir no mínimo o ensino fundamental completo e ser destro (confirmado a partir do teste de dominância manual de Edinburgh)^(14,15)^. Como critérios de exclusão, definiu-se: apresentar queixas auditivas; alteração de orelha média; queixas e/ou sinais e sintomas neuropsiquiátricos e/ou alterações de fala perceptíveis.

Considerando esses critérios, participaram da pesquisa 50 sujeitos normo-ouvintes, destes 39 do sexo feminino (78%) e 11 do sexo masculino (22%), com idades entre 19 e 40 anos, com média de idade de 25,5 anos.

Já para o grupo de sujeitos com perda auditiva foram considerados os seguintes critérios de inclusão: apresentar idade maior que 19 anos; perda auditiva do tipo condutiva, sensorioneural ou mista^(16)^; limiares de audibilidade médios das frequências de 500, 1000, 2000 e 4000 Hz, variando de perda auditiva de grau leve à moderada^(17)^; apresentar resultado no Índice Percentual de Reconhecimento de Fala (IPRF) em ambiente silencioso entre 100 e 80%; apresentar queixa auditiva de dificuldade de reconhecimento de fala no ruído, possuir no mínimo o ensino fundamental completo e ser destro (confirmado a partir do teste de dominância manual de Edinburgh)^(14,15)^. Estabeleceu-se como critérios de exclusão: queixas e/ou sinais e sintomas neuropsiquiátricos; alteração cognitiva (triada por meio do Mini Exame de Estado Mental)^(18,19)^ e/ou alterações de fala perceptíveis.

Participaram do estudo, 12 sujeitos com perda auditiva, sendo estes 5 do sexo feminino (41,67%) e 7 do sexo masculino (58,33%), com idades entre 38 e 70 anos e média de 58,42 anos. Destes sujeitos, 1 orelha apresentava perda auditiva do tipo condutiva (4,76%), 17 orelhas perda sensorioneural (80,95%) e 3 orelhas perda mista (14,29%). Já em relação ao grau da perda auditiva, 9 orelhas apresentavam perda leve (42,86%) e 12 orelhas grau moderado (57,14%).

##### Instrumentos e procedimentos

Os participantes foram submetidos à anamnese direcionada, com perguntas referentes aos dados pessoais, nível de escolaridade, história otológica e queixas auditivas. Posteriormente foi realizada a inspeção visual do Meato Acústico Externo (MAE) de ambas as orelhas, avaliação das medidas de imitância acústica, Audiometria Tonal Liminar (ATL), logoaudiometria, e finalmente foram avaliados com o instrumento proposto neste estudo.

As medidas de imitância acústica foram realizadas utilizando-se o imitanciômetro da marca *Interacoustics* modelo AT 235. A realização da ATL e a aplicação do instrumento de avaliação proposto, foram executadas utilizando-se o audiômetro da marca *Interacoustics,* modelo AC 33, e fones auriculares, modelo TDH 39, em ambiente tratado acusticamente. Além de um *Compact Disc Player*, da marca Toshiba, modelo CD-4149, acoplado ao audiômetro, para apresentação dos estímulos de fala e do ruído, em gravação digital. A duração média da avaliação completa de cada sujeito foi de 60 minutos.

##### Análise dos dados

Para investigar a validade de critério e determinar os valores de referência para o TPR foi utilizada a técnica da Curva ROC (*Receiver Operating Characteristic*).

Inicialmente, foi realizada a comparação entre os desempenhos das orelhas avaliadas (orelha direita e esquerda) dos sujeitos normo-ouvintes. Não foram observadas diferenças estatisticamente significantes na variável orelha para os vocábulos monossilábicos (p=0,463) e dissilábicos (p=0,295) por meio do teste de Wilcoxon.

Posteriormente, foi realizada a comparação de desempenho entre sujeitos normo-ouvintes do sexo feminino e masculino. Também não foram observadas diferenças estatisticamente significantes na variável sexo para os vocábulos monossilábicos (p=0,242) e dissilábicos (p=0,171) no teste de Mann-Whitney.

Estas verificações possibilitaram que os valores de referência fossem gerados considerando o resultado geral das orelhas, totalizando 100 orelhas avaliadas, gerando um ponto de corte único independente destas variáveis, a fim de facilitar a interpretação dos resultados.

Para a realização das análises estatísticas, foram utilizados os programas SPSS V20, Minitab 16 e Excel Office 2010. Foi considerado resultado significante p ≤ 0,05, com confiança de 95%.

## RESULTADOS

### Etapa 1: Material resultante

O instrumento proposto neste estudo foi intitulado Teste de Palavras no Ruído (TPR). O conteúdo do teste inclui uma faixa com o sinal de referência de 1 KHz (tom puro) e o ruído com espectro de fala para fins de calibração, uma faixa com a frase introdutória de instrução do teste, uma lista treino monossilábica, duas listas equivalentes monossilábicas, apresentadas com duas sequências de distribuições diferentes das palavras e cinco listas equivalentes, dissilábicas.

Considerando que são apenas duas listas de palavras monossilábicas, e buscando evitar o efeito memória/aprendizagem em aplicações diversas, foram disponibilizadas duas sequências diferentes de distribuição das palavras, formando quatro listas (L1 sequência 1 e L1 sequência 2; L2 sequência 1 e L2 sequência 2).

As listas de palavras e o ruído foram gravados em canais independentes, permitindo assim que os níveis de apresentação em cada canal fossem ajustados de forma isolada.

A frase introdutória de instrução de execução do teste no ruído, é a seguinte: “Você vai ouvir uma lista de palavras e um ruído. Ignore o ruído e repita cada palavra ouvida do jeito que entender”.

Em todas as listas os vocábulos são precedidos da ordem carreadora “repita a palavra”.

### Etapa 2: Estratégia de aplicação do instrumento selecionada

A partir da observação das diferentes estratégias de aplicação executadas nos cinco indivíduos, análise dos dados e relato dos participantes após a realização do teste e também, baseado na literatura, optou-se como estratégia de aplicação do TPR nesta pesquisa, a obtenção do LRF na presença de um ruído fixo.

Para a obtenção das medidas, o TPR foi aplicado por meio de fones auriculares, de forma monoaural, sendo que o estímulo de fala foi apresentado juntamente com o ruído. Para que o nível de apresentação dos dois estímulos pudesse ser ajustado de forma independente, estes foram gravados em canais diferentes. Assim, antes de iniciar a aplicação do teste, o equipamento foi calibrado usando o *VU-meter,* ajustando a saída de cada canal no nível zero, usando como referência um tom puro de 1 kHz presente no mesmo canal em que estavam gravadas as palavras, enquanto que no outro canal, foi usado o próprio ruído do teste, por ser um ruído contínuo.

As listas mono e dissilábicas, foram aplicadas de forma randomizada, e as medidas foram obtidas tomando o cuidado de avaliar as orelhas de forma alternada, classificando os sujeitos em pares e ímpares, e assim a avaliação dos sujeitos pares foi iniciada pela orelha direita e dos sujeitos ímpares, iniciada pela orelha esquerda.

Desta forma, o TPR foi aplicado de acordo com a seguinte ordem de apresentação:

1. Calibração independente de cada canal do equipamento;
2. Instrução sobre a estratégia de resposta do teste no ruído no lado da orelha selecionada para iniciar o teste;
3. Aplicação da lista treino apresentando as 10 primeiras palavras monossilábicas, com a presença de ruído competitivo, para familiarizar o sujeito com o teste, alternando a orelha;
4. Aplicação de uma lista de monossílabos, na presença do ruído competitivo, alternando novamente o lado da orelha;
5. Aplicação de uma lista de dissílabos, na presença do ruído competitivo, mantendo o lado da orelha avaliada anteriormente;
6. Aplicação de uma lista de monossílabos, na presença de ruído competitivo, alternando o lado da orelha;
7. Aplicação de uma lista de dissílabos, na presença do ruído competitivo, mantendo o lado da orelha avaliada anteriormente.

Para a pesquisa do LRF utilizando o TPR, foi usada a estratégia sequencial ou adaptativa, ou ainda ascendente-descendente^(20)^, que possibilita determinar a condição na qual o indivíduo é capaz de reconhecer em torno de 50% dos sinais de fala ouvidos.

Para iniciar a pesquisa do LRF no ruído, foi utilizada uma relação S/R de + 10 dBNA e apresentada a lista treino. Com base nisso, foi iniciada a aplicação do teste, apresentando a primeira palavra da lista em um nível de 10 dBNA acima do valor do primeiro erro cometido durante a aplicação da lista treino, o que garante que o sujeito inicie acertando a primeira palavra da lista, buscando assim diminuir a variabilidade das medidas e servindo também como motivador para o sujeito que está sendo avaliado. Na sequência, a próxima palavra foi apresentada em um nível de 4 dBNA abaixo e assim sucessivamente, até o sujeito avaliado apresentar a primeira resposta incorreta, e a partir daí, foram usados intervalos de apresentação de 2 dBNA, até o final da lista, de acordo com a resposta do sujeito, ou seja, quando a resposta foi incorreta, foi aumentado 2 dB, quando foi correta, foi diminuído 2 dB^(20)^.

A estratégia de resposta solicitada foi a repetição das palavras da forma que entendessem, e nos casos nos quais o sujeito respondeu duas palavras semelhantes, por ter ficado com dúvida, considerou-se a primeira palavra repetida.

Para a obtenção dos LRFs, foram anotados os níveis de apresentação de todas as palavras e calculadas as médias destes valores a partir do valor no qual ocorreu a primeira reversão de resposta (primeiro erro), até o final da lista.

Considerando que uma relação S/R é obtida pelo cálculo da diferença, em dB, entre o valor do LRF (média dos níveis de apresentação da fala na presença de um ruído) e o valor do ruído competitivo utilizado, este cálculo foi realizado, a partir dos valores obtidos para cada lista, subtraídos do nível de apresentação do ruído utilizado nesta pesquisa, que foi de 55 dBNA.

O tempo estimado de aplicação do TPR é de 30 segundos para calibração, 11 segundos para execução da frase de instrução, em torno de 1 minuto e 30 segundos para aplicação da lista treino, 3 minutos e 22 segundos para execução da lista monossilábica em cada orelha, e 3 minutos e 20 segundos para aplicação das listas dissilábicas em cada orelha. Assim sendo, o tempo total aproximado para a execução do teste em ambas as orelhas com monossílabos ou com os dissílabos é de 9 minutos, e se necessário, 16 minutos para aplicação das listas mono e dissilábicas associadas.

### Etapa 3: Validade de critério e valores de referência

Na Tabela 1, pode ser observada a comparação entre o desempenho das orelhas dos sujeitos normo-ouvintes e orelhas dos sujeitos com perda auditiva no TPR, na avaliação com os vocábulos mono e dissilábicos. Tanto para o estímulo monossilábico quanto para o dissilábico, foram verificadas diferenças estatisticamente significantes entre os grupos avaliados, sendo observado melhor desempenho no grupo de normo-ouvintes. Também é possível observar que tanto os sujeitos normo-ouvintes quanto os sujeitos com perda auditiva, apresentaram melhor desempenho quando avaliados com os vocábulos dissilábicos.

**Tabela 1:** Comparação de desempenho por orelha entre sujeitos normo-ouvintes e com perda auditiva no Teste de Palavras no Ruído

		**N**	**Média**	**Mediana**	**Desvio Padrão**	**Q1**	**Q3**	**IC**	**P-valor**
Monossílabos	Normo-ouvintes	100	-2,38	-2,39	1,86	-3,82	-1,17	0,36	<0,001*
	Perda Auditiva	21	6,44	6,17	4,31	3,78	9,83	1,85	
Dissílabos	Normo-ouvintes	100	-5,99	-6,15	1,63	-7,23	-4,70	0,32	<0,001*
	Perda Auditiva	21	1,61	0,81	2,70	-0,36	4,14	1,16	

Teste Mann-Whitney. Resultados expressos em relação Sinal/Ruído

* Valor estatisticamente significante no nível de 5% (p ≤ 0,05)

**Legenda:** N: número de orelhas; Q: quartil; IC: Intervalo de confiança

Na análise da curva ROC, verificou-se que ambas as curvas são estatisticamente significantes com p-valor <0,001 e valores de *Area Under the Curve (*AUC) extremamente altos, sendo 0,987 e 1,000, respectivamente, para os estímulos monossilábicos e dissilábicos.

As curvas ROC para os monossílabos e dissílabos são apresentadas nas Figuras 1 e 2, respectivamente.

**Figura 1:** Curva ROC para o Teste de Palavras no Ruído com Monossílabos

**Figura 2:** Curva ROC para o Teste de Palavras no Ruído com Dissílabos

Na Tabela 2 estão expressos os pontos de corte para o TPR monossilábico e dissilábico. Os pontos de corte ideais foram determinados encontrando os valores que permitiram o melhor equilíbrio entre sensibilidade e especificidade.

**Tabela 2:** Pontuação de corte do Teste de Palavras no Ruído obtido por meio das curvas ROC, e correspondente sensibilidade e especificidade

	**Ponto de corte**	**Sensibilidade**	**Especificidade**
Monossílabos	1,47	90,5%	98,0%
Dissílabos	-2,02	100,0%	99,0%

Ponto de corte expresso em relação Sinal/Ruído

## DISCUSSÃO

A proposta do TPR surgiu da necessidade de complementação da bateria audiológica básica, com um teste de reconhecimento de fala no ruído de rápida e fácil aplicação, com características psicométricas bem definidas. O teste apresenta a função de investigar uma das principais queixas auditivas relatadas pelos sujeitos ao realizar a avaliação auditiva na prática clínica, que é a dificuldade de reconhecer a fala em ambientes ruidosos^(3)^.

A estratégia de aplicação do TPR definida para esta pesquisa, que foi a pesquisa do Limiar de Reconhecimento de Fala (LRF) utilizando o ruído fixo em 55 dB NA, buscou representar uma situação comunicativa comumente vivenciada no dia a dia.

O LRF quando apresentado com ruído competitivo, é capaz de mensurar o desempenho do reconhecimento de fala, e estabelecer a relação S/R necessária para o indivíduo repetir corretamente 50% dos estímulos de fala apresentados no ruído^(21)^.

De acordo com o relatório da Organização Mundial de Saúde^(22)^, medidas de ruído ambiental acima de 55 dB(A), já podem provocar estresse leve, dando início a comportamentos como desconforto auditivo, estado de vigilância e agitação, o que pode provocar alterações nos resultados de um teste subjetivo. Tal informação reforça a escolha do nível de apresentação utilizado nesta pesquisa, visando minimizar o cansaço e desconforto auditivo do sujeito avaliado.

Esta estratégia de avaliação é amplamente aplicada em testes internacionais que avaliam o reconhecimento de fala no ruído utilizando palavras como estímulo, tais como, *Words in Noise Test* (WIN)^(23)^, *Digits in Noise* ^(24)^ e *The Speech in Babble (SiB)* ^(25)^. Também é recomendada por diretrizes profissionais, que consideram o procedimento adaptativo de pesquisa do LRF no ruído, como o mais adequado para ser utilizado na bateria de avaliação audiológica básica, o qual fornece uma medição rápida e padronizada^(26)^.

A principal vantagem da utilização do LRF como estratégia de avaliação do reconhecimento de fala no ruído, é o fato do nível de apresentação do estímulo de fala ser adaptável de acordo com a resposta de cada sujeito, evitando assim o efeito teto (ceiling *effect*) e efeito solo (*floor effect*) do teste, os quais estão relacionados com pontuações extremamente superiores (100%) e inferiores (0%), respectivamente^(27)^.

Quanto à comparação entre o desempenho no TPR dos sujeitos normo-ouvintes e dos sujeitos com perda auditiva e queixa de dificuldade de reconhecimento de fala no ruído, foi observado melhor desempenho dos sujeitos normo-ouvintes, tanto na avaliação com estímulo monossilábico quanto dissilábico (Tabela 1), evidenciando a validade de critério. Este achado vai ao encontro de outros estudos que igualmente avaliaram o reconhecimento de fala no ruído por meio de testes com palavras^(3,28)^ e também observaram melhor desempenho dos sujeitos normo-ouvintes que não apresentavam queixa relacionada ao reconhecimento de fala no ruído. Ainda, foi possível perceber que ambos os grupos apresentaram melhor desempenho quando avaliados com os vocábulos dissilábicos (Tabela 1). Este resultado já era esperado, pois quanto maior a extensão de um vocábulo, mais fácil é seu o reconhecimento^(29)^.

A análise da curva ROC, revelou evidências satisfatórias de validade de critério por meio de valores de AUC extremamente altos (Figura 1 e 2), o que significa que o TPR, apresentou adequada capacidade de distinção entre os sujeitos normo-ouvintes e sem queixa de dificuldade de reconhecer a fala no ruído e os sujeitos com perda auditiva e com queixa de dificuldade de reconhecer a fala no ruído.

Quanto aos valores de referência (ponto de corte) estabelecidos para o TPR, os resultados da análise da sensibilidade e especificidade (Tabela 2) indicaram a relação S/R de 1,47 para o TPR-M (90,5% de sensibilidade, 98,0% de especificidade) e de -2,02 para o TPR-D (100,0% de sensibilidade, 99,0% de especificidade). Para este estudo foi considerada como sensibilidade a capacidade do teste em identificar sujeitos com dificuldade de reconhecimento de fala no ruído, já a especificidade foi considerada a capacidade do teste expressar resultados dentro da normalidade, para aqueles sujeitos que não apresentam dificuldade para reconhecer a fala no ruído.

Portanto, desempenhos em relações S/R mais favoráveis (mais positivas), do que esses valores, demonstram que o sujeito avaliado apresenta dificuldade de reconhecimento de fala no ruído e indicam a necessidade da utilização de outros materiais e testes complementares para uma avaliação mais detalhada. Estes testes devem permitir dimensionar melhor o quanto esses resultados alterados predizem, sobre as dificuldades comunicativas enfrentadas no dia a dia por determinado indivíduo, como por exemplo, avaliação com estímulo de sentenças e/ou avaliação das habilidades do processamento auditivo.

Quanto ao estímulo a ser utilizado para a avaliação do reconhecimento de fala no ruído, baseado nas observações realizadas no presente estudo, acredita-se que ambos os estímulos aqui apresentados (vocábulos monossilábicos e dissilábicos) podem ser utilizados, mas é importante considerar que cada um trará diferentes informações, podendo ser usados inclusive conjuntamente para elucidar melhor a capacidade do indivíduo para reconhecer a fala no ruído na rotina clínica.

Sabe-se que as palavras monossilábicas, apesar de carregarem contexto linguístico, estão mais relacionadas à audibilidade. Já as palavras dissilábicas, carregam contexto linguístico maior e fornecem um número superior de pistas auditivas, o que, como pode ser confirmado nesta pesquisa, facilita o reconhecimento das mesmas, em relações S/R mais desfavoráveis de escuta, e também são mais representativas do dia a dia, em função do maior número de dissílabos no português brasileiro.

Assim, considerando isso, pode-se sugerir que o avaliador aplique preferencialmente as listas monossilábicas, e então, quando o sujeito apresentar desempenho insatisfatório (ou aquém do esperado), seja então aplicada uma lista de dissílabos, com o objetivo de verificar o quanto o indivíduo é capaz de aproveitar o aumento das pistas semânticas e linguísticas, proporcionadas pelo aumento da extensão das palavras, na tarefa de reconhecer a fala^(30)^.

Para o resultado da aplicação do TPR-D associado ao TPR-M, é esperada uma relação S/R no mínimo igual e idealmente inferior (mais desafiadora) ao avaliar os sujeitos com os vocábulos dissilábicos. O não aproveitamento do aumento das pistas auditivas e linguísticas proporcionadas pelos dissílabos podem indicar alterações de diferentes habilidades de processamento auditivo e/ou cognitivas, às quais necessitam de uma investigação mais detalhada.

O TPR demonstrou ser aplicável na rotina clínica audiológica, rápido e de fácil utilização e interpretação, além de ter sido desenvolvido na língua Portuguesa Brasileira, constituído por palavras familiares, ruído com espectro de fala e ser apresentado em formato digital. Também apresentou capacidade de identificação de sujeitos com dificuldade de reconhecimento de fala no ruído, tanto com o estímulo monossilábico, quanto dissilábico.

Desta forma, sugere-se a inserção do TPR, na bateria de avaliação audiológica básica daqueles sujeitos com perda auditiva de grau leve a moderado^(17)^, ou perda auditiva com configuração descendente, que apresentam adequada ou discreta dificuldade de reconhecimento de fala no silêncio, avaliado por meio da logoaudiometria convencional, porém referem queixas relacionadas ao reconhecimento de fala na presença de ruído, a fim de detectar de forma objetiva esta queixa.

Ao utilizar o TPR, o examinador deve estar atento ao grau e configuração da perda auditiva a fim de assegurar que o ruído esteja sendo percebido, considerando o fato de que o nível de apresentação do ruído no TPR é de 55 dBNA, garantindo assim que a avaliação esteja sendo realizada na presença de ruído competitivo.

Destaca-se que caso o examinador use diferentes níveis de ruído, ou faça qualquer mudança na estratégia de aplicação do teste, os valores de referência descritos neste trabalho não serão válidos.

Sobre as limitações do estudo, em função do tempo determinado para essa pesquisa, não foi possível aplicar o TPR em sujeitos com perdas auditivas mais acentuadas, o que necessitaria de algumas adaptações no protocolo de aplicação, sendo uma possibilidade de estudo futuro.

## CONCLUSÃO

O TPR foi proposto para utilização na bateria audiológica básica, a fim de identificar sujeitos com dificuldade de reconhecer a fala no ruído. Foi definida como estratégia de aplicação do teste a pesquisa do Limiar de Reconhecimento de Fala com ruído fixo em 55 dBNA. O TPR demonstrou ser rápido e de fácil aplicação e interpretação dos resultados, podendo ser uma ferramenta útil a ser utilizada na rotina clínica audiológica. Além disso, apresentou evidências satisfatórias de validade de critério, capacidade de identificação de sujeitos com dificuldade de reconhecimento de fala no ruído, tanto com o estímulo monossilábico, quanto dissilábico. Foram definidos como valores de referência os pontos de corte expressos em relação S/R de 1,47 dB para o TPR-M e de -2,02 dB para o TPR-D.
